# Essential Management of Pediatric Brain Tumors

**DOI:** 10.3390/children9040498

**Published:** 2022-04-02

**Authors:** Katharina Lutz, Stephanie T. Jünger, Martina Messing-Jünger

**Affiliations:** 1 Neurosurgery Department, Inselspital, 3010 Bern, Switzerland; 2 Pediatric Neurosurgery, Asklepios Children’s Hospital, 53757 Sankt Augustin, Germany; m.messing@asklepios.com; 3 Center for Neurosurgery, Department of General Neurosurgery, Faculty of Medicine and University Hospital Cologne, University of Cologne, 50937 Cologne, Germany; stephanie.juenger@uk-koeln.de

**Keywords:** pediatric neurosurgery, pediatric gliomas, medulloblastoma, ependymoma, targeted therapy, individualized tumor-treatment

## Abstract

Brain tumors are the most common solid tumors in children and are associated with high mortality. The most common childhood brain tumors are grouped as low-grade gliomas (LGG), high grade gliomas (HGG), ependymomas, and embryonal tumors, according to the World Health Organization (WHO). Advances in molecular genetics have led to a shift from pure histopathological diagnosis to integrated diagnosis. For the first time, these new criteria were included in the WHO classification published in 2016 and has been further updated in the 2021 edition. Integrated diagnosis is based on molecular genomic similarities of the tumor subclasses, and it can better explain the differences in clinical courses of previously histopathologically identical entities. Important advances have also been made in pediatric neuro-oncology. A growing understanding of the molecular-genetic background of tumorigenesis has improved the diagnostic accuracy. Re-stratification of treatment protocols and the development of targeted therapies will significantly affect overall survival and quality of life. For some pediatric tumors, these advances have significantly improved therapeutic management and prognosis in certain tumor subgroups. Some therapeutic approaches also have serious long-term consequences. Therefore, optimized treatments are greatly needed. Here, we discuss the importance of multidisciplinary collaboration and the role of (pediatric) neurosurgery by briefly describing the most common childhood brain tumors and their currently recognized molecular subgroups.

## 1. Introduction

Brain tumors are the most common solid tumors in children and are associated with high mortality [1,2]. The most common childhood brain tumors are classified as low-grade gliomas (LGG), high grade gliomas (HGG), ependymomas, and embryonal tumors according to the World Health Organization (WHO) [3,4,5,6,7]. Radiation exposure is the only environmental factor shown to be associated with an increased risk of brain tumor development [1].

Various brain tumor registries (such as the National Program of Cancer Registries [NPCR] and the Surveillance, Epidemiology, and End Results [SEER] Registry) provide population-based data from patients with central nervous system tumors. These data can be used to analyze brain tumors on the basis of histology, location, age, survival, clinical features, and other characteristics [8,9]. According to combined data analysis, from 2008 to 2017, the incidence rates for malignant brain tumors and other central nervous system (CNS) tumors in children and adolescents increased from 0.5% to 0.7% per year, whereas those in all other age groups decreased [8]. Malignant brain tumors are observed more frequently from 1 to 4 years of age. Low-grade brain tumors are common until infancy and further increase in incidence until adolescence. The 5-year relative survival rate for malignant brain tumors is 77% on average in children younger than 14 years of age and 81% in those 15–19 years of age. The 5-year relative survival of patients with non-malignant brain tumors is almost 100% (98 and 99%) in both age groups [8].

Advances in molecular genetics have led to a shift from a pure histopathological diagnosis to integrated diagnosis, which was first included in the WHO classification published in 2016 [6] and further updated in the 2021 edition [7]. Integrated diagnosis is based on molecular genomic similarities of tumor subclasses, which can better explain the different clinical courses of previously identical histopathological entities. This new subclassification may ideally reveal new therapeutic targets for individual tumor therapies. 

In the past decade, many advances have been made in diagnostics, molecular genetic pathology, surgical techniques, and non-surgical therapeutic methods. For several pediatric tumors (e.g., medulloblastoma and LGG), these advances have significantly improved therapeutic management and prognosis in certain subgroups. For other tumors (e.g., HGG), the prognosis remains dismal despite these advances. Several therapeutic approaches also have serious long-term consequences. Thus, optimized treatment and development of new therapeutic methods are greatly needed. In particular, advances in molecular biology have moved the field toward the goal of an individualized therapy. For some tumors, these innovations have already influenced treatment modalities; for other tumors, promising therapeutic agents remain to be developed [2].

Here, we discuss the importance of multidisciplinary collaboration and the role of (pediatric) neurosurgery by briefly describing the most common childhood brain tumors and their currently recognized molecular subgroups.

## 2. Clinical Assessment/Symptoms

In medicine, children cannot simply be considered small adults, and a specialized multidisciplinary team is mandatory to manage all treatment related aspects in children with CNS tumors [1]. Pediatric brain tumor related symptoms and signs can be unspecific, thus delaying diagnosis. Therefore, several brain tumor awareness programs have been initiated [10,11]. In infants and young children, not only the tumor itself but also an associated hydrocephalus can significantly impair neurodevelopment and have severe long-term sequelae. Neurological signs and symptoms in pediatric brain tumors can be different compared to adult patients. It is recommended that pediatric patients should be seen by a pediatric neurologist and/or neurosurgeon with extensive experience in pediatric neuro-oncology (ideally by a pediatric neurosurgeon). No clinical correlation exists between tumor type and symptoms. Clinical and radiological tumor presentations vary significantly and are dependent on the age of the child and the tumor growth patterns [12].

Children with brain tumors may present with focal symptoms [1]. These symptoms depend on the exact location of the tumor and the adjacent structures. Motor and sensory deficits may occur, for example, if the central cortex or corticospinal tracts are affected. A language deficit can result from impairment of the language centers or the connections of these regions (frontoparietotemporal left-sided tumors, e.g., Broca’s, Wernicke’s, or fasciculus arcuatus). Cerebellar tumors often result in ataxia, fine motor dysfunction, and balance disorders. However, cranial nerve deficits may also be observed, and, depending on the extent of brainstem impairment, respiratory and circulatory disorders may also occur [13].

Focal symptoms may be accompanied by further symptoms due to increased intracranial pressure (ICP). The increased intracerebral pressure might be caused by the tumor mass or concomitant hydrocephalus. Again, the symptoms often differ among age groups. For example, signs of elevated ICP in infants may manifest as a tense fontanel, sunset phenomenon, and vomiting. In addition, non-specific symptoms, such as macrocephaly, irritability, failure to thrive, or loss of developmental milestones, can be signs of increased ICP caused by a brain tumor or associated hydrocephalus. In older children, high ICP may present with headache, nausea and vomiting, anisocoria, impaired vision, and decreased vigilance [1]. For a subset of tumors growing in the suprasellar region, signs of endocrine dysfunction should also warrant suspicion.

In addition, attention should be drawn to family history and a possible syndromic disease. Several syndromes with elevated risk of developing a brain tumor during childhood, such as neurofibromatosis type 1 (NF-1) and type 2 (NF-2), tuberous sclerosis, Li-Fraumeni syndrome, and Gorlin syndrome [1]. 

Seizures can be directly induced by a supratentorial brain tumor [13] or a high ICP due to associated hydrocephalus. In infants, failure to thrive may be the only sign of a brain tumor.

Whenever a child presents with neurologic symptoms, acute ICP should always be considered, and immediate admission to a specialized clinic should be sought, because any delay in diagnosis and treatment is potentially life-threatening.

## 3. Diagnostics 

After the clinical examination and detailed anamnesis by the general pediatrician and/or pediatric neurologist, and possibly a (pediatric) neurosurgeon, any subtle suspicion of a pediatric brain tumor should be investigated with sliced imaging as early as possible. The diagnostic gold standard for a suspected brain tumor is magnetic resonance imaging MRI [14]. A larger mass can also be seen in computed tomography (CT) scans. CT is justified in emergencies, such as a comatose child presenting with dilated pupils who requires immediate surgery. However, because of its radiation exposure and limited diagnostic value, CT should be used in children only in emergency settings or when an MRI is not feasible. In the case of eloquent neighboring regions, functional MRI (fMRI) can be applied to reveal the involvement of important brain areas. Special approaches, such as diffusion studies, can be used to determine tumor type [15,16]. The MR spectroscopy (MRS) can additionally contribute to the differentiation of the tumor entity [17]. 

Some of the tumors (e.g., medulloblastoma, pilocytic astrocytoma, or ependymomas) are associated with leptomeningeal or intrinsic dissemination. If suspected, to confirm the diagnosis, an MRI of the entire neuroaxis should be acquired. Furthermore, cerebrospinal fluid (CSF) samples can be obtained through lumbar puncture to determine leptomeningeal spreading after exclusion of a potential risk of brain herniation [18,19]. In cases of suspected germ cell tumors, the assessment of serum and CSF tumor markers is crucial for the initial treatment stratification [20]. Because most brain tumors are associated with elevated ICP or may involve optic structures, ophthalmological assessment is recommended. In suprasellar midline tumors, additional hormonal status determination is advisable.

Because of the importance of rapid and complete work up, a pediatric neurologist, pediatric oncologist and/or (pediatric) neurosurgeon should be involved immediately after hospital admission. This multidisciplinary management at the beginning of treatment ensures immediate and appropriate treatment according to the existing protocols available for most of the tumor entities.

## 4. Clinical Approach and Therapy 

To ensure an optimum treatment strategy in the management of children with brain tumors, a multidisciplinary team of specialized clinicians is mandatory. The disciplines involved must be coordinated at regular intervals. Each individual discipline has its area of expertise and performs its assigned role. However, decisions are often made and supported collectively by several or even all the involved disciplines. The exact treatment approach to treating children with brain tumors is described in the following.

Initially, the pediatrician frequently has the leading role. In cases of suspected brain tumors, a specialist (pediatric neurologist and/or (pediatric) neurosurgeon) should be consulted from the beginning of treatment. If signs of elevated ICP are present, the immediate involvement of a (pediatric) neurosurgeon is mandatory, to enable early emergency interventions and avoid unnecessary delays. After neuroimaging, discussion with a neuroradiologist is necessary to narrow down the most likely differential diagnosis.

The subsequent approach depends on the symptoms and the suspected diagnosis of the child. If the suspicion of elevated ICP is confirmed on imaging, emergency intervention has to be considered because of the associated life-threatening risks. Therapy includes optimized body positioning (e.g., neck elevation at 30° to avoid jugular vein compression), short-term hyperventilation [21], administration of steroids [22] and hyperosmolar therapy (e.g., mannitol, or hypertonic saline) [21], and more invasive procedures, such as the insertion of an external ventricular drain, insertion of an Ommaya reservoir for serial puncture, endoscopic ventriculostomy, or emergency tumor resection. In the absence of elevated ICP, subsequent procedures are decided jointly by collaborators from different disciplines. As a rule, an internal multidisciplinary discussion among a pediatrician, pediatric neurologist, pediatric oncologist, radiation oncologist, (pediatric) neurosurgeon, and nursing staff [23] is important. The group composition may vary and must be adapted to each individual case. In the further course, other disciplines will be added to the multidisciplinary team. A multidisciplinary neuro-oncology group also includes a (neuro-) psychologist and/or physiotherapist. The leading department is defined, and subsequently performs coordination tasks and serves as direct contact for the family. Often, pediatric oncology or pediatric neurology is the clear leading department choice. However, neurosurgery may be chosen, depending on the individual situation. The possible suspected diagnosis is discussed, and the further procedure are determined step by step as follows.

The (pediatric) neurosurgeon indicates the possible surgical procedures, e.g., whether a tumor resection is possible or whether a biopsy can instead be attempted from a surgical view-point. Depending on the suspected diagnosis, the pediatric oncologist emphasizes the extent of resection. Several scenarios can be collaboratively reviewed. Inclusion in ongoing clinical studies must be considered by the oncologist. The (neuro-) psychologist might already better address fears and psychological issues, and support the family.

After multidisciplinary discussion, the family should be informed of all possible consequences of the disease. The first joint conversation with the family is usually conducted by a member of one of the disciplines, and complementary information is provided by the other specialists. The family is introduced to all the involved disciplines and is carefully guided through the conversation with the help of a (neuro-) psychologist. Together, the multidisciplinary team can best provide all important information to the family and the patient. Children must be included in this communication in an age appropriate manner. They tend to feel guilty about their parents’ confusion or sadness, and can better cope with situations when they know the underlying reasons. This knowledge also minimizes anxiety and stress. Sometimes showing images of the tumors to patients is helpful, even if they are young. Depending on the situation, the further procedure is then be discussed in more detail with the parents alone. The (pediatric) neurosurgeon explains and illustrates the possible surgery. Questions can be answered in detail. The presentation of needed postoperative therapies may also be important. Here, depending on the psychological condition of the family, the information must be coordinated. The presence of a (neuro-) psychologist is particularly important, so that the family can be supported from the beginning of the process, and any fears will be made apparent to the (neuro-) psychologist and can be considered.

The initial consultation also sets the time schedule. The subsequent procedures and the difficult conditions for the family are clarified, including surgery, postoperative surveillance in the intensive care unit, waiting for the pathology results, and planning the further adjuvant treatments (e.g., chemo- and radiation therapy), if needed. Possible neurological deficits, necessary therapies (physiotherapy, speech therapy, and occupational therapy), and, if needed, neurological rehabilitation should also be discussed. This meetings at the beginning of the process, addressing all contingencies, can help families feel that they are not left alone. In these discussions, the function of each discipline (particularly surgical, adjuvant, and supportive) acting together in the multidisciplinary treatment team must be explained. Recurring joint meetings with physicians and therapists, as well as nursing staff, are part of the care of these children and families.

After the family is briefed, the timing of surgery is determined. The timing depends on the urgency of the operation (e.g., signs of elevated ICP) and the optimal conditions required in the operating theater. The optimal team consists of at least an experienced neurosurgical team (ideally including a pediatric neurosurgeon), and a pediatric anesthesiologist with experience in neuro- as well as pediatric anesthesia [22], and a neurophysiologist for intraoperative neurophysiological monitoring (IONM) [24]. Total venous anesthesia (TIVA) is preferred, not only in surgeries under IONM. In suprasellar lesions, additional hydrocortisone stress prophylaxis is needed [22]. Intraoperative cooperation among a (pediatric) neurosurgeon, pediatric oncologist, and neuropathologist is also essential. The tumor tissue is made available to the neuropathologist for intraoperative histopathological analysis. The neuropathologist must be available during surgery for preliminary frozen section diagnosis, which can be discussed in direct telephone consultation with the (pediatric) neurosurgeon and consequently influences the course of the operation [25]. Notably, the intraoperative diagnosis is not a final diagnosis but merely indicative. For definitive histopathological and molecular diagnosis, a larger total amount of tissue may be required. In most countries, a final reference pathology is obligatory. After frozen section diagnosis, if resection is difficult because of anatomical conditions, a pediatric oncologist may be consulted during surgery to determine whether more radical resection might be particularly important. 

Immediate postoperative care should be provided by an experienced team of pediatric and neurosurgical intensivists. Close monitoring is performed in the pediatric intensive care unit for the safety of the patient. If the clinical course is stable, the patient can be transferred to the leading discipline’s normal ward the next day. Mobilization is usually accomplished with the assistance of physical therapy. Evaluation is performed to identify even the smallest neurological deficits to initiate the most effective therapy (physiotherapy, speech therapy, and/or occupational therapy). Regular wound checks are performed by a (pediatric) neurosurgeon.

Contrast MRI is typically performed in the first 72 hours after surgery to assess the resection status and rule out CSF circulation disorders or possible complications. A resectable tumor remnant should always be discussed for second look surgery by the multidisciplinary team [26,27]: Tumor entities respond differently to adjuvant therapies. For chemosensitive tumors, a pediatric oncologist may offer therapy. If residual tumor is seen on MRI, tumor re-resection must be discussed if the tumor does not respond well to chemotherapy. A radiation oncologist may also recommend re-resection. For example, for residual tumors near eloquent brain regions, tumor volume reduction should be considered, to decrease the radiation dose and spare the eloquent areas.

Radiation oncologist and pediatric oncologists often work together and jointly monitor the further course of therapy, according to tumor entity and the response to therapy. Depending on the age of the child, a time-saving therapy with chemotherapeutic agents can be performed first, so that radiation therapy is not started until the child’s brain is more mature [28]. Another collaborative decision is whether chemotherapy should be administered concomitantly with radiation therapy. When intrathecal administration of chemotherapy is required, the indication for an Ommaya reservoir should be made by a pediatric oncologist and a (pediatric) neurosurgeon.

Until discharge, the involved disciplines discuss new developments and prepare the family as best as possible for the prognosis. Rehabilitation needs are evaluated, including aspects of (neuro-) psychology, physiotherapy, speech therapy, and ergotherapy. The recommended adjuvant treatments are explained to the family and, if necessary, integrated into the course of the intended rehabilitation or scheduled as a separate inpatient or outpatient stay. To optimize the quality of treatment, follow-up examinations (imaging and clinical) are often performed in collaboration among several disciplines (e.g., pediatric oncology, radiation oncology, and (pediatric) neurosurgery, with the involvement of (neuro-) psychology). For all major aspects, the case is re-presented to the tumor board and the best possible treatment options can be re-evaluated. Contacting the general treating pediatrician directly may be helpful to organize further follow-up after discharge. He plays a critical role in supporting the families (e.g., with any decision-making), and good collaboration with the pediatric oncologist is very helpful.

(Neuro-) Psychological care must be provided for not only acute situations, but also long-term consequences. The diagnosis of a brain tumor can cause stress for children and their families. The therapies required may also induce stress. For example, chemotherapy can cause neurocognitive and neuropsychological late effects, even 10 years after treatment. Risk factors, such as lower physical activity, have been identified and can aid in determining the therapeutic approach. Various strategies can be applied to counteract the effects of stress. Psychological support plays a central role, as does physical activity. Post-traumatic stress disorder is also increasingly found in parents of children with brain tumors, which in turn also affects the children [29]. The main goal of psychological care during hospitalization is to prevent psychological stress and to initiate supportive therapy at an early stage to improve quality of life and functioning [29].

### 4.1. The Role of Neurosurgery

The extent of tumor resection remains the most important factor with respect to event free and overall survival in nearly all kinds of tumor types [30]. Resection of a tumor-mass can provide immediate relief from tumor-related signs and symptoms, and improve long-term outcomes and survival. Additionally, the tissue obtained during surgery is used to provide a histopathological and molecular-genetic diagnosis. For this purpose, an adequate amount of tumor tissue must be sampled. Depending on the localization and histology, gross total resection (GTR) and minimum morbidity ensuring maximum quality of life, are the goal of the (pediatric) neurosurgeon [24,30,31]. In case of significant remnant or recurring tumors, second look surgery, must be considered, if feasible and depending on tumor entity [26,27]. The new management concepts of pediatric brain tumors underscore the importance of these second look surgeries and the removal of metastases.

The surgical anatomy of pediatric brain tumors differs substantially from those in adult patients. Highly eloquent midline structures, such as the basal ganglia, brainstem, and cerebellum are most often involved. Intraventricular and suprasellar involvement are also common locations. The frequent involvement of midline structures and the posterior fossa explains a high rate of associated hydrocephalus. Primary treatment stratification therefore always includes hydrocephalus management if necessary. Shuntplacement or endoscopic third ventriculostomy are indicated if tumor resection does not resolve the CSF disorder [30]. Maximally safe resection in these regions is demanding and requires a high expertise. Surgery by dedicated pediatric neurosurgeons ensures the better outcomes than interventions performed by inexperienced surgeons [27,32].

The safety of the child should always be the focus. To achieve a maximum resection without harming the child, various tools are available to (pediatric) neurosurgeons. For surgical preparation, microsurgical instruments and a microscope are used. Depending on the location of the tumor and the preference of the (pediatric) neurosurgeon, neuronavigation can help plan the approach. The navigation also contributes to the extent of resection. Integration of additional fMRI information may also contribute to complete removal from a functional perspective [30]. Intraoperative imaging methods can be used for tumor localization and to verify the extent of resection; examples include real-time ultrasound [30,33,34] and intraoperative MRI [35,36]. Intraoperatively performed MRI images can also be fed into the navigation systems. As an additional tool, an endoscope can reach deeper or hidden areas without a large access route, thus improving resection results, such as in craniopharyngiomas [37]. Both eloquent brain areas and cranial nerves can be detected and controlled by IONM [38,39]. IONM can help preserve brain function and is indispensable in the resection of deeply located brain tumors, e.g., in the posterior fossa as well as of spinal cord tumors [30,40]. This method has been demonstrated to be safe and reliable in children of all ages [30,31].

Biopsy may be required when tumor resection is not feasible because of high morbidity or mortality, or when a specific entity, such as a diffuse intrinsic pontine glioma (DIPG), is suspected. For superficial tumors, an open biopsy is possible. For deep-seated tumors, a stereotactic procedure is most suitable, because it provides high safety with low morbidity [41]. In certain tumors, e.g., neoadjuvant chemo- or radiation therapy can be an appropriate treatment after biopsy and tumor marker assessment and represents another interface of multidisciplinary collaboration [42].

New methods in neuropathology (see Section 4.4 and Section 5.1) have led to further subdivision and subtypes of known tumor entities, included in the new tumor classifications of the WHO [4,5,6,7]. (Table 1).

### 4.2. The Role of Radio-Oncology

Radiation therapy is the next important component in the multidisciplinary treatment of many brain tumors in children, particularly for aggressive tumor types [43]. The radiation strategy depends on the tumor type and subtype, and sometimes on patient age. Radiation therapy in young children has a significant negative influence on the neurocognitive development and also affects the hormonal systems and other body regions. In embryonal tumors, such as medulloblastoma, adjuvant radiation therapy of the whole brain and spine is recommended after resection, as well as an additional boost dose to the tumor bed [43]. In children younger than 3–5 years, radiation therapy is avoided when possible.

To decrease radiotoxicity in the healthy brain and adjacent body structures, fractionated or hyperfractionated radiation therapy has become a standard in pediatric brain tumor treatment. Treatment protocols usually consist of daily radiation sessions over 6 weeks until the target volume dose is reached. To ensure a reproducible and accurate patient positioning during radiation application, the head is fixed with a mask. With this mask, CT images of the head are produced, and the target volume is calculated. Critical organs at risk (such as brain stem or optic nerves) are also determined and spared as much as possible [43]. Because the resolution of the CT is insufficient, a cranial high-resolution MRI readout should also be fused with the CT images to increase the accuracy of detecting the target volume and organs at risk [43]. Special MRI simulators have the advantage of generating MRI images in the desired treatment position, and the inaccuracy associated with fusion of image-sets in different positions is minimized [43,44]. Data on MRI-only radiation treatment planning are available, and this method is expected to become more important in the future, particularly in the treatment of children [43,44].

Because of the vulnerability of the developing brain, there is a high risk of significant late effects, particularly if combination therapies, such as radiation therapy and chemotherapy, are administered [45]. Children younger than 3–5 years may have severe and irreversible late effects, such as neurocognitive damage, growth arrest, or development of secondary tumors [45]. Efforts are being made to improve the effectiveness and simultaneously decrease the high toxicity of radiation therapy. More precise images can help with treatment planning. Newer radiation therapy techniques, such as intensity-modulated radiation, volumetric-modulated arc therapy, particle beam therapy and stereotactic radiation, can better protect the surrounding healthy tissue [43]. 

Photon-based radiation therapy methods, such as volumetric-modulated arc therapy, have also made progress in recent decades and enabled high speed treatment and thus better tolerability in children [43]. Conventional fractionated radiation therapy continues to be used for many different brain tumors in children [46]. For diagnoses with a very short overall survival, such as diffuse midline gliomas, hypofractionated radiation therapy can be applied because of its relatively shorter treatment time and smaller treatment burden [47].

Particle therapy (proton beam therapy) shows similar efficacy to photon radiation therapy but decreases the risk of adverse effects [45]. This method is mainly used in young patients with expected late sequelae, a high chance of survival, and, in the case of a high target volume, an increased risk of neighboring structures [48]. Preliminary data on tumor control, progression-free survival, and cognitive decline are available [45]. Proton beam radiation is frequently used for germ cell tumors, ependymomas, LGG, medulloblastomas, chordomas and craniopharyngiomas [45]. Stereotactic radiosurgery consists of numerous focused photon beams creating a high precision target volume. This technique is considered for single session high dose radiation therapy; although fractionation is also possible (stereotactic radiation therapy), this technique is rarely used in children. In ependymoma protocols, a stereotactic system can be used for boost radiation [43].

### 4.3. The Role of the Neuro-Oncology

Pediatric oncologists have a variety of tools at their disposal, including “classical” chemotherapy, targeted therapies, anti-angiogenic therapies, and novel procedures such as Tumor Treating Fields (TTFs). Most first and second line treatments follow specific protocols according to national or international guidelines. With the expansion of molecular-genetic tumor analysis, a trend towards individualized therapy in pediatric brain tumors is underway. (Table 2).

#### 4.3.1. Chemotherapy

Chemotherapy is a keystone in neuro-oncology, targeting dividing tumor cells [45]. Administration modes include oral administration, intravenous administration, local administration into the tumor bed, and intrathecal administration via a ventricular reservoir [61,62]. Various chemotherapeutic agents, such as alkylators, platinum compounds, or etoposide (topoisomerase II blocker), and intrathecal methotrexate (folic acid antagonist) are used. Numerous studies have examined chemotherapeutic agents for different tumor entities, to investigate their efficacy as standalone therapies or in combination with radiation therapy. Neoadjuvant administration or high-dose chemotherapy with myeloablative effect and stem cell rescue are also under investigation [63]. The recommendation for chemotherapy and the type of chemotherapeutic agents are subject of tumor-related treatment protocols. In very young children with highly radiosensitive tumors, high-dose chemotherapy is used until the age of 3–5 years when radiation therapy is less toxic to the developing brain [5]. (Table 2).

#### 4.3.2. Immunotherapy

Immunotherapy is an additional therapeutic neuro-oncology tool to enhance the patient’s own antitumor immune response. Promising methods include immune checkpoints (with monoclonal antibodies), chimeric antigen receptor T therapy, oncolytic viruses, and vaccine therapy [64,65]. Trials in adults are considered feasible and safe. However, ongoing studies are aimed at providing evidence of prognostic benefit. Data from adults cannot be directly applied to the treatment of pediatric brain tumors, first, because the immune system of the pediatric central nervous system functions differently, and second, because of the distinctive genetic and epigenetic nature of pediatric brain tumors [65].

#### 4.3.3. Anti-Angiogenic Therapy

Another therapeutic approach is anti-angiogenic therapy. By inhibiting angiogenesis, tumor growth can be directly influenced, and the effects of other therapeutic agents can be indirectly improved [41]. However, tumors have mechanisms to counteract anti-angiogenic therapy. One of the best established agents is bevacizumab, a humanized monoclonal antibody that inhibits VEGF activity. In some pediatric tumors (e.g., LGG and medulloblastoma), a partial imaging response and symptom improvement has been demonstrated; in other tumors (e.g., HGG and ependymomas), the effect has been minimal [66]. Combination therapy with a chemotherapeutic and a cytostatic agent has demonstrated good tolerability and a partial response in HGG [67].

#### 4.3.4. Tumor-Treating Fields

TTF is a new form of therapy that has been successful in the treatment of glioblastoma in adults [68]. Alternating electric fields with low intensity (<2 V/cm) and moderate frequencies (100–300 kHz) induce an inhibitory effect on cell division when applied to the scull via transducer arrays. Ongoing studies with TTFs in pediatric patients are primarily evaluating the toxicity, and safety, efficacy, and benefits of TTF in children with supratentorial HGG and ependymomas [69].

### 4.4. The Role of Neuropathology and Molecular Neuropathology

The role of the neuropathologist has changed over time and with new developments. In addition, essential differences exist between pediatric and adult brain tumors in terms of growth pattern and histological and molecular genetic features [5,70]. (Table 1).

According to the suspected tumor type, tumor tissue in the form of formalin-fixed, paraffin-embedded tissue and fresh specimens is sent to the neuropathologist. The tissue is used once for frozen sectioning and final histological examination, and a portion of the sample is processed for molecular analysis. Intraoperative consultation with a neuropathologist is recommended when diagnostic feedback to the operating (pediatric) neurosurgeon may influence the course of surgery [25]. Other information such as the patient’s age, imaging findings, demographics, and medical history, as well as the suspected intraoperative diagnosis, additionally contribute to the diagnosis [25]. 

The histopathological diagnosis of brain tumors is made on the basis of light microscope, according to the neuroanatomical location of the tumor and the morphological nature of the tumor cells. The morphology of the tumor cells are compared to specific cells of the still developing or already mature normal brain cells [25,71]. The tissue must be treated so that further (also molecular) analysis is feasible [25]. The findings are complemented by various analyses, such as immunohistochemical and cytogenic markers, which help identify additional subgroups according to the expression patterns of specific cell line markers and identified abnormalities [7,71]. Cytology provides cellular detail and tissue architecture and can be performed with various staining methods. The phenotype is determined according to histological features (e.g., evidence of mitotic activity, microvascular proliferation, or presence of necrosis) [25].

The potential analysis of genomic, transcriptomic, and epigenomic features leads to a more accurate classification of brain tumors, and thus enabling new treatment options and prognostic assessments [71]. In particular, tumors that could not be distinguished by conventional methods can now be categorized into different tumor subgroups. Genomic features can be revealed by next generation sequencing (NGS) with automated deoxyribonucleic acid (DNA) sequencing [71], thus enabling the exact DNA signature of tumors to be visualized. Transcriptomic profiling can reveal RNA-based abnormalities that are undetectable with genetic methods (e.g., also using NGS) [71,72]. Epigenetic pathways are responsible for the regulation of gene expression. In tumors, mechanisms such as DNA methylation and histone modification can lead to the inactivation of tumor suppressor genes or the activation of oncogenes, and thus influencing tumorigenesis. DNA methylation profiles can be used to detect these alterations [71].

These new methods are leading to further subdivision of known tumor entities and may result in new targeted therapeutic approaches in pediatric neuro-oncology [73]. New targeted therapy options are increasingly being clinically tested and integrated into treatment protocols [2,7]. (Table 2).

## 5. Molecular Advances and Their Effects on Clinical Management

### 5.1. Integrated Diagnosis

Growing insight into the genetic and epigenetic background of childhood CNS tumors has changed the histology-based classification of CNS tumors and led to the definition of new tumor classifications and subtypes, as reflected by the regularly updated tumor classifications of the WHO [4,5,6,7]. (Table 1). Diagnosis includes various investigative information and is presented as an integrated diagnosis, which combines tissue-based histological and molecular diagnosis and is quantified in a layered report with histological diagnosis, CNS WHO grade, and molecular information [4,7]. Some tumors are described in general terms. For accurate final integrative diagnosis, the molecular profile is needed to divide tumors into subgroups [4,6,7]. (Table 1).

New molecular methods have improved the understanding of certain brain tumor groups. Previously, tumors thought to be of embryonic origin were classified as primitive neuroectodermal tumors. However, DNA methylation profiling has revealed that tumors within that former classification belonged to entirely different brain tumor groups. This new classification can explain the observed differences in clinical courses, and enables more appropriate therapy to be applied [71,74].

The classification of tumor types into further subgroups can enable more individualized therapy overall. On the one hand, more accurate prognostication of tumors is possible, so that the therapy can be adapted accordingly. For tumors with better prognosis, for example, a de-escalation of the therapy regime can be evaluated. Second, the molecular-genetic markers provide a basis for the development of new tumor-specific agents to improve tumor control and overall outcome [45].

Below, to provide an update on the current state of the art in treating pediatric neuro-oncology patients, we summarize the recent diagnostic advances in the most frequent pediatric brain tumors and their effects on the clinical multidisciplinary management.

### 5.2. Medulloblastoma

Because medulloblastoma is the most common malignant brain tumor in children [75], many children with this brain tumor are also treated by general pediatricians. Therefore, familiarity with the broad clinical spectrum of this disease is important. Medulloblastoma belongs to the group of embryonal tumors [4,7] and accounts for almost 10% of all pediatric brain tumors [18]. Two age peaks can be observed, one at 3–4 years and the other between 8 and 10 years [18]. Hereditary cancer predisposition syndromes associated with medulloblastoma, such as familial adenomatous polyposis (FAP) and Gorlin syndrome, have been described but account for less than 5% [18]. 

Therapy consists of maximum tumor resection and, depending on age and risk stratification, cranio-spinal irradiation and maintenance chemotherapy [5,73]. (Table 2). The role of the (pediatric) neurosurgeon is complete tumor resection of the medulloblastoma, whenever possible, because GTR or near-complete resection is associated with a better prognosis [1]. After the final diagnosis of medulloblastoma, close collaboration between radiation oncologists and pediatric oncologists is highly valuable. To delay radiation therapy, given its highly toxic effects, high-dose chemotherapy is administered to children below 3–5 years of age [1,5]. (Table 2).

Histopathologically, a medulloblastoma is typically classified as a small blue cell tumor, but other morphological patterns are seen as well [76]. Medulloblastomas were previously classified according to histopathological features into four morphological groups: desmoplastic/nodular, MBEN (medulloblastoma with extensive nodularity), classic medulloblastoma, and large cell/anaplastic medulloblastoma [7,76]. Risk stratification was based on age, the extent of resection, metastases and histology [77]. Because prognoses differed in tumors with the same risk classification, improved diagnostic strategies were needed. New diagnostic technologies, such as NGS and DNA methylome profiling, have resulted in a new tumor classification of medulloblastomas [7,73,76], according to the concept that an analogous molecular transcriptome corresponds to similar tumor behavior [75,76]. 

In 2010, four principle medulloblastoma subgroups were defined by transcriptional profiling ((i) Wingless [WNT], (ii) Sonic Hedgehog [SHH], non-WNT/non-SHH: (iii) group 3, and (iv) group 4) [76,77]. Each has a specific tumor biology and prognosis, thus leading to new risk stratification schemes [75]. These findings were first included into the 2016 WHO classification of central nervous system tumors [6], as an integration of histopathology and molecular diagnosis, and were further specified in the new 5th WHO classification in 2021 [4,7]. Because of the wide variation in prognosis, a brief summary of the four major subgroups is provided. (Table 1).

**Figure 1:**
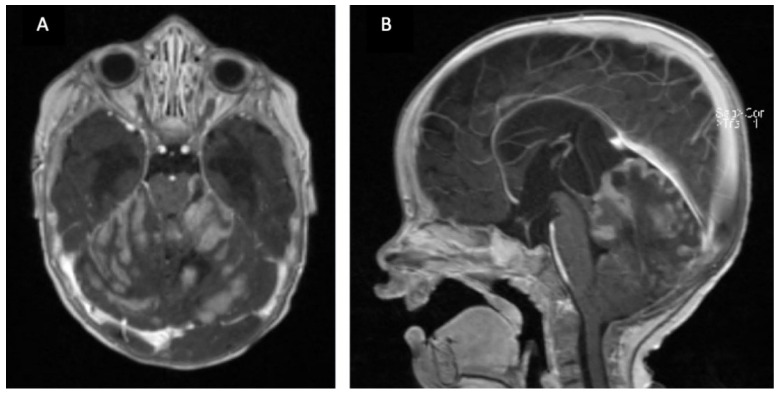
6 month old boy with macrocephaly and developmental delay. Contrasted T1 MRI (**A**,**B**) with large cerebellar tumor and associated hydrocephalus. Diagnosis: SHH-medulloblastoma with extensive nodularity (MBEN) WHO grade 4. Gorlin syndrome (SUFU mutation).

**Figure 2:**
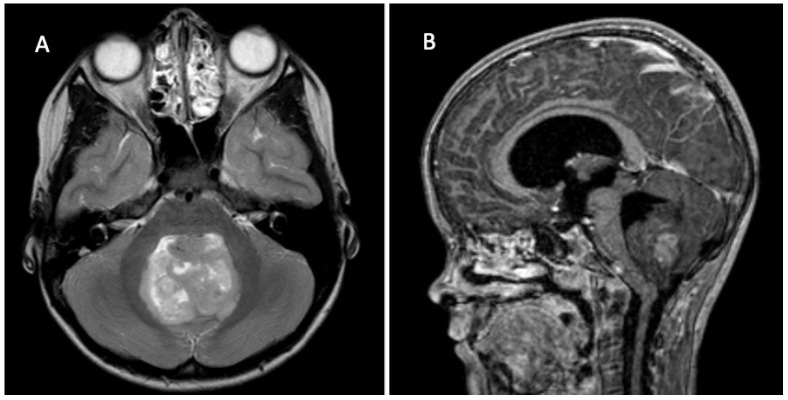
7 year old girl with signs of raised intracranial pressure and ataxia. (**A**) Axial T2 MRI shows midline tumor in the posterior fossa with IVth ventricle compression. (**B**) Sagittal contrasted T1 MRI with partially enhancing tumor and associated hydrocephalus. Diagnosis: Medulloblastoma, non-WNT/non-SHH (WHO grade 4).

(i) WNT [7]. In this subgroup, the WNT pathways, which play roles in cell cycle control and embryogenesis, are activated. The subgroup has only few genomic alterations [1,77] and is determined by the occurrence of mutations in the gene beta-catenin and the simultaneous presence of monosomy 6 [5]. WNT medulloblastomas tend not to occur in infancy, but may be present at any other age [76]. Patients younger than 16 years of age have a very good prognosis after resection and radiation therapy of the craniospinal axis. Even in residual or metastatic tumors, a low risk profile is likely for WNT tumors in children. Adult patients have a slightly higher risk [77]. WNT medulloblastomas typically show a classic histology. 

(ii) SHH, TP53-mutant and TP53-wildtype [7]. In the SHH subgroup, the SHH signaling pathway is activated. In some of the tumors, amplification of MYCN and GLI2 and mutations of TP53 are also found. Children younger than 3 years of age most often show activation of the SHH pathway. If a TP53 mutation is detected, the risk is higher, and the prognosis is poorer; such mutation is particularly common in children 3–17 years of age. Younger children have a lower risk. Overall, the prognosis is moderate. Compared with the other subgroups, SHH tumors show more frequent local recurrences [77]. SHH activation is associated with Gorlin syndrome, and genetic testing and family counseling are recommended. A desmoplastic histology has the most favorable prognosis in this subgroup. (Figure 1).

(iii) Group 3 [7]. Group 3 shows recurrent MYC amplification. Isochromosome 17 q, activation of GFI1A/GFI1B, and OTX2 amplifications are also found. Metastases are frequent, and the overall prognosis is worse than that of other subgroups, particularly when MYC amplification is detected. Recurrences with metastatic dissemination occurs most frequently in this group [77]. Group 3 medulloblastoma affects frequently infants and children, but rarely adults [76]. (Figure 2). 

(iv) Group 4 [7]. There are different characteristics, such as isochromosome 17q, MYCN amplification, duplications of SNCAIP, or loss of 11q and others. This is the most common subgroup, and it occurs mainly in children and adolescents [77]. (Figure 2).

A large cell and anaplastic (LCA) histology is mostly associated with unfavorable outcomes. WNT tumors and non-metastatic group 4 tumors with complete loss of chromosome 11 or complete gain of chromosome 17 are associated with a favorable prognosis (survival > 90%).

These discoveries may lead to changes in treatment in the near future. De-escalation of existing treatment protocols is under discussion, with the goal of minimizing potential long-term adverse effects [1,77]. High-risk groups (survival 50–75%) include patients with tumors from the metastatic SHH subgroup or group 4 and SHH subgroup with MYCN amplifications. The very high risk group (survival < 50%) includes patients with tumors from group 3 with metastases and the SHH subgroup with TP53 mutation [77]. Currently, Hedgehog-pathway blockers are under clinical investigation. Unfortunately, an acquired resistance against the smoothened (SMO) (Table 2) inhibitor Vismodegib (GDC-0449) has been observed [5,78]. Other targeted therapies are being tested, such as GLI blockers or agents against the PI3K or CDK inhibitor pathway, as well as immunotherapeutic drugs [79].

To assess the most appropriate diagnosis and support further research, the (pediatric) neurosurgeons should always send sufficient (fresh) tumor material to a neuropathologist. A trend towards resecting metastatic and recurrent tumors for the same reasons.

### 5.3. Glioma

#### 5.3.1. Low-Grade Glioma 

Approximately 30% of all pediatric brain tumors are LGGs. Most of them tend to grow slowly, and patients may have a long history of symptoms. Acute symptoms can also occur, owing to associated hydrocephalus or rapid growth of tumor cysts. The cerebellum is the most prevalent location (15%–25% of all pediatric brain tumors), followed by the cerebral hemisphere (10%–15%), the basal ganglia (10%–15%), optic pathways (5%), and the brain stem (2%–4%). Approximately 15 to 20% of children with neurofibromatosis type 1 develop optic pathway/hypothalamic glioma [80].

**Figure 3:**
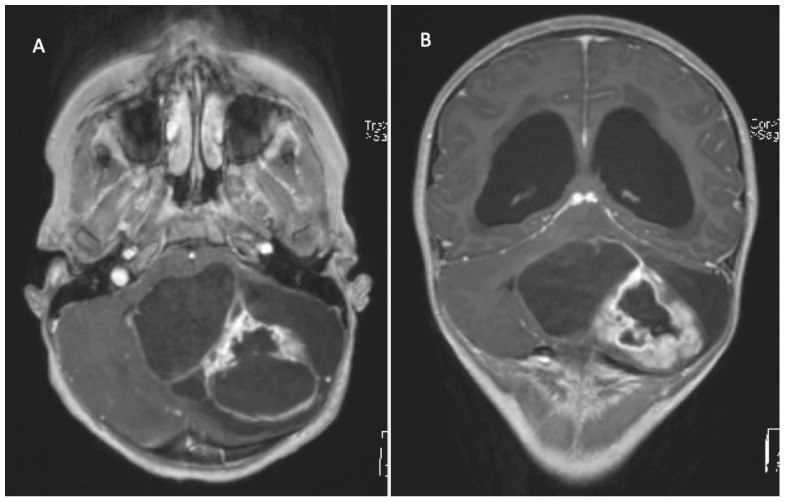
12 year old girl with acute signs of raised ICP and schooling difficulties. Contrasted T1 MRI shows large cystic tumor with contrasting solid components and chronic hydrocephalus (**A**,**B**). Diagnosis: pilocytic astrocytoma (WHO grade 1).

LGGs consist of grade 1 and 2 tumors according to the WHO classification, and frequently show cerebellar localization [1,4,5,6,7]. Pilocytic astrocytoma (Figure 3) is the most common representative, but other astrocytic tumors, subependymal giant cell astrocytomas, pleomorphic xanthoastrocytomas, oligodendroglial tumors, and benign neuroepithelial tumors are also observed [5,7]. (Table 1).

Again, the (pediatric) neurosurgeon’s role is to completely resect the tumor, if possible, because GTR may be curative for LGGs, particularly for pilocytic astrocytomas [1,5]. (Table 2). The roles of radiation oncologists and pediatric oncologists are particularly important in cases of non-resectable tumors. Chemo- or radiation therapy may be applied, depending on the clinical course and patient age [1]. (Table 2). For suspected LGG in highly eloquent locations, e.g., the supra-sellar region or incidental lesions, a wait-and-see approach may also be discussed, because rapid progression or malignant transformation are rarely seen [81]. In a wait-and-see approach, close monitoring by the multidisciplinary team, including the general pediatrician, is recommended. (Table 2).

After incomplete resection, recurrence is common, but the prognosis is often very good [5]. Re-resection should be discussed and performed if feasible. Some tumors are age related, such that proliferation stops during adulthood.

In addition, in LGGs, the challenge for the neuropathologist is to provide an integrated diagnosis (Table 1). One of the evolving mechanism in pediatric LGG is the disruption of the RAS-mitogen-activated protein kinase (MAPK) pathway, which leads to the BRAF V600E mutation or the BRAF:KIAA1549 fusion gene. Alterations in the BRAF oncogene are frequently found in pilocytic astrocytomas. Other alterations in RAS/MAPK pathways and FGFR mutations may also occur [1,4,5,7,82]. IDH1/IDH2 mutations are very rare in pediatric astrocytomas WHO grade 2, and a specific age related entity must be discussed, because malignant transformation is seen more rarely in children than adults [1].

The general pediatrician must also be aware that a different therapeutic regimens should be performed in patients with LGG and NF1. These patients often show a disease course that is not very aggressive [5]. As many as 15% of children with NF1 develop low-grade gliomas of the visual pathways, whereas other brain regions are less often affected (3–5%). NF1 gliomas are frequently associated with the loss of functional neurofibrin, resulting in the activation of the oncoprotein RAS [82,83]. These tumors are typically asymptomatic and do not require therapy; in some cases, they may even regress spontaneously. Chemotherapy should be given if clinical deterioration, e.g., visual loss, occurs. However, children younger than 2 years are at higher risk of tumor growth and death. Aberrations in the RAS/MAPK pathway or other transcriptional regulators are often observed. Biopsy should be discussed in rapidly growing tumors to identify more aggressive subtypes presenting with loss of ATRX or CDKN2A/p16 [84].

The new WHO 5th classification of central nervous system neoplasms includes four entities in the pediatric-type low grade diffuse glioma group [4,7]. (Table 1). According to histopathology and molecular characterization, the integrated diagnoses are sub classified as (i) diffuse astrocytoma, MYB- or MYBL1 altered, (ii) angiocentric glioma, (iii) polymorphous low-grade neuroepithelial tumor of the young, and (iv) diffuse low-grade glioma, MAPK pathway-altered [4,7]. Unlike the non-diffuse tumors, diffuse LLG are often very difficult to address surgically, because of their location (midline, deep-seated), and diffuse nature; consequently, adjuvant treatment must be discussed by the multidisciplinary team [5].

Targeted therapy is increasingly being investigated in LGGs and must be considered by pediatric oncologists. Some clinical experiences in pediatric LGGs treated with BRAF or MEK 1/2 inhibitors have been published (Table 2), and the first clinical trials on NF-1-associated LGG are ongoing [5]. In addition, FGFR-targeted agents and other tyrosine kinase inhibitors are being studied [82]. The National Cancer Institute-Children’s Oncology Group Pediatric MATCH trial aims to combine various targeted therapies (NCT03155620).

To precisely assess the tumor type and gain more insights into the molecular-genetic background of LGG, biopsy in primarily inoperable lesions is becoming more important, including in NF-1-related neoplasms. Additionally, a possible target for alternative treatment options can be identified.

#### 5.3.2. High-Grade Glioma and Midline Glioma

Children with HGG usually have a short history and are likely to have symptoms suggestive of increased ICP. These patients are also often admitted as emergencies, and diagnosis must be assessed as quickly as possible.

According to the WHO, grade 3 and 4 tumors are high-grade gliomas [4,6,7]. (Table 1). Histologically, they are often similar or identical to adult tumors, but their clinical course and molecular markers significantly differ from those in adults. Therapy usually consists of maximum resection and local radiation therapy. Additional chemotherapy is usually included into treatment protocols, although whether major benefits are provided remains unclear. Temozolomide, CCNU, vincristine, or a combination therapy are mainly used [1]. Some tumors, e.g., DIPG or basal ganglia tumors, cannot be sufficiently resected, because of their diffuse nature and deep localization [1]. (Table 2). In pediatric supra-tentorial HGG, TTF may also be applied [69]. Case series have shown a partial response and good tolerability [85].

In HGG, specific molecular features with prognostic significance have been identified. Histone 3 mutations in diffuse intrinsic pontine and basal ganglia glioma are associated with a dismal prognosis. Inactivation of tumor protein 53 (TP53) is also a negative outcome predictor as well. To date, no therapeutic approach has been found to address the underlying (epi)genetic aberrations, and the prognosis of children with HGG has not yet significantly improved [5].

**Figure 4:**
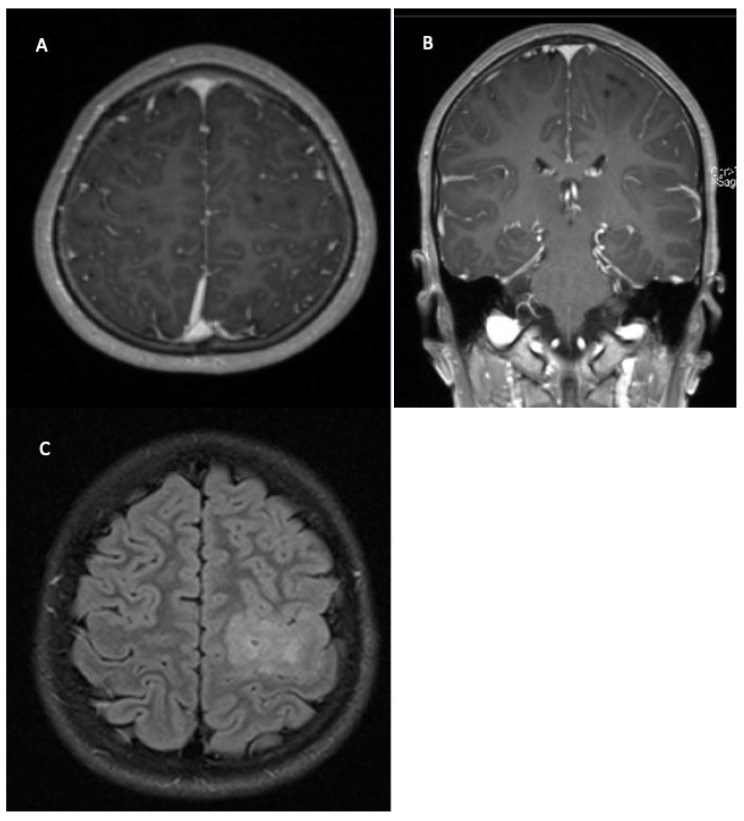
13 year old boy with right hand paresis and initial focal seizure. Contrasted T1 MRI shows non-enhancing cortical tumor (**A**,**B**), FLAIR sequence with diffuse cortical lesion (**C**). Diagnosis: diffuse hemispheric glioma, H3 G34-mutant (WHO grade 4). Therapy: Near total resection, conformal radiation therapy, temozolomide, tumor treating fields (TTF).

Four different diffuse childhood HGG types have been defined according to the current WHO classification: (i) diffuse midline glioma, H3 K27-altered, (ii) diffuse hemispheric glioma, H3 G34-mutant (Figure 4), (iii) diffuse pediatric-type high-grade glioma, H3-wildtyp, and (iv) IDH-wildtype and infant-type hemispheric glioma [4,7,86]. These diagnoses are based on histopathological and molecular findings. The term “glioblastoma” is no longer common in pediatric HGG [4,7]. (Table 1).

One of the most aggressive tumor entities of this group, the diffuse midline glioma, most often occurs in the pons (DIPG) and is typically diagnosed by MRI and clinical signs of progressive brainstem dysfunction [1]. Tumor resection is not feasible because of the deep localization and associated morbidity [87]. The standard protocol consists of radiation and chemotherapy, although the prognosis does not significantly improve after treatment. Under clinical trial conditions, stereotactic biopsy is recommended by many pediatric oncologists [88]. (Table 2). This treatment is associated with low surgical risk and morbidity, and should facilitate understanding of the nature of this deadly diagnosis and identifying alternative treatment options in the future [1,88]. Genomics-based treatment target identification is part of many ongoing studies and appears promising [87].

### 5.4. Ependymoma

**Figure 5:**
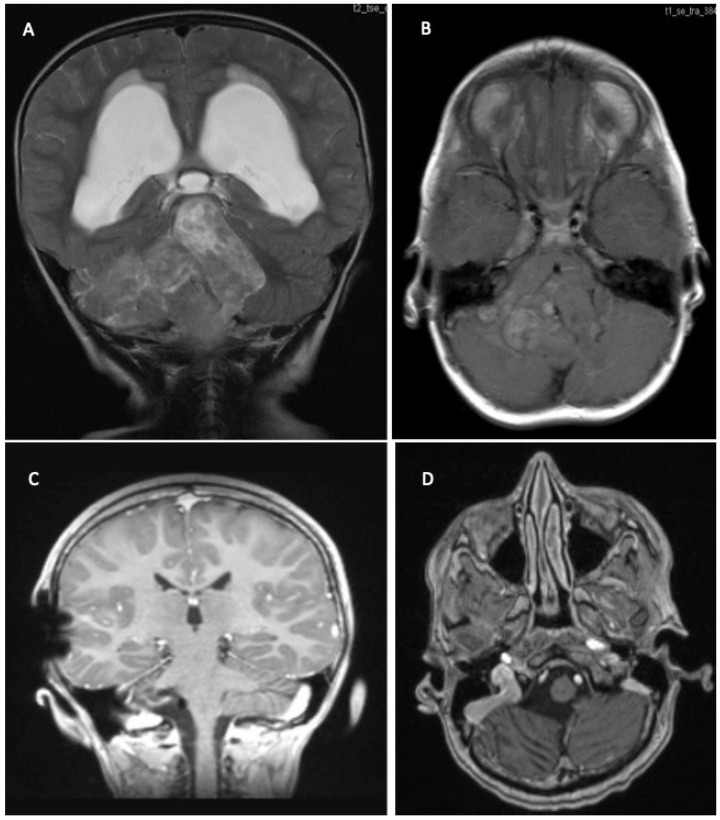
2 year old boy with psychomotor delay and signs of raised intracranial pressure. T2 (**A**) and contrasted T1 MRI (**B**) shows large posterior fossa tumor and associated hydrocephalus. Diagnosis: ependymoma without anaplasia (no molecular diagnostic) and shunt implantation. Therapy: GTR (**C**,**D**) followed by chemo- and conformal radiation therapy. Complete remission for 12 years.

In children, both cranial and spinal symptoms may be present, because pediatric ependymomas occur along the entire neuraxis and account for 10% of all pediatric brain tumors [89]. Intracranial manifestation, particularly in the posterior fossa, is more frequent than spinal tumors in children [90,91]. (Figure 5). Ependymomas were previously considered one entity with different tumor grades. WHO grading continues to be used in recent and ongoing studies, but age- and location-specific risk-stratification according to biologically distinct subtypes, appears more adequate [92,93,94,95,96,97,98].

The new WHO classification defines distinct ependymoma subtypes [4,7]. (Table 1). Supratentorial ependymomas are divided into ST ependymomas with ZFTA (former RELA-/C11orf95–fusion positive ependymomas), tumors with a YAP1-MAMLD1 fusion [4], or other rare fusions [99]. The most frequent, RELA-fusion positive tumors (now ZFTA), do not far worse in the pediatric population [100,101,102]. A YAP1-MAMLD1 fusion occurs in young children and shows an excellent prognosis even after surgical resection alone [92,95,96,102], whereas a deletion/inactivation of CDKN2A is a negative prognostic parameter [100,103].

**Figure 6:**
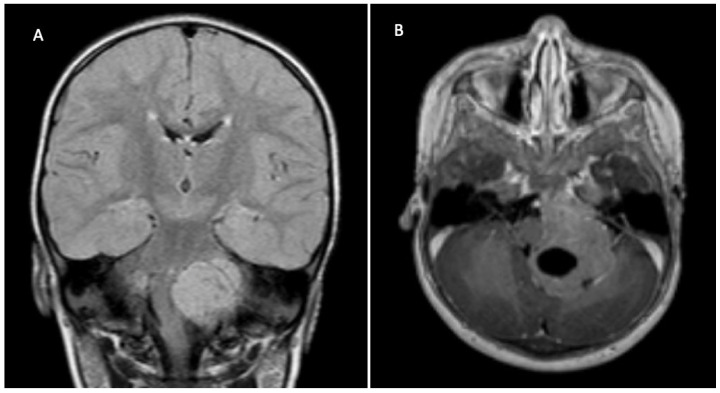
11 months old girl with swallowing difficulties and facial palsy. FLAIR MRI (**A**) and contrasted T1 MRI (**B**) shows enhancing tumor invading the cerebellum from the left cerebellopontine angle. Diagnosis: anaplastic ependymoma, PFA-type. Therapy: after NTR chemotherapy (HIT-MED Therapy Guidance modified SKK) and proton-beam irradiation, early local recurrence during adjuvant treatment and re-resection, 3rd look surgery for tumor residual after radiation therapy termination.

Infratentorial ependymomas are divided into PF type A (PFA) and PF type B (PFB) ependymomas. PFA ependymomas have an overall balanced genome with a gain of chromosome 1q in approximately 20% [95,98,104], and a CpG-island hypermethylation [94,95,98], and show little or no H3K27me3 expression [105,106], thus enabling diagnosis by immunohistochemistry. They affect younger children and are associated with a poor prognosis. (Figure 6).

PFB ependymomas affect older children and adults, and have hypomethylated genomes, polyploid chromosomal profiles, and retained H3K27me3 [94,95,98,105,106]. They have a favorable prognosis and may be cured by surgery alone.

Spinal ependymomas are rare and primarily affect older children [90,91]. They are classified as myxopapillary ependymoma WHO grade 2, formerly WHO grade 1, which was changed, because of their clinical course in the new WHO classification, to classic (WHO grade 2), and anaplastic ependymoma (WHO grade 3) [4,7]. The presence of a MYCN amplification indicats poorer prognosis [4,107,108].

To date, data are insufficient to allow individual molecular groups to be assigned WHO grades. 

Regarding treatment, maximally safe surgery, including repeated surgery, has the strongest prognostic effects [28,100,101,102,104,109]. Most tumors require adjuvant radiation therapy [28,109], which may be performed from the age of 12 months onwards [28,101,102,109]. (Table 2).

The role of chemotherapy remains debatable to date and is currently being analyzed in ongoing trials (e.g., SIOPEII-Trial). (Table 2). Patients with PF-B or YAP1-fusion positive ependymoma may be cured by surgery alone [101,102]. The same applies in completely resected WHO grade 2 spinal ependymomas [110].

Finally, given the frequent late recurrences, long-term follow-up is warranted [100].

## 6. Future Perspectives

Important advances have been made in pediatric neuro-oncology during the past decade. A growing understanding of the molecular-genetic background of tumorigenesis has improved the diagnostic accuracy, e.g., with respect to prognosis and defining distinct tumor subgroups. Re-stratification of treatment protocols and the development of targeted therapies will significantly influence the overall survival and quality of life of our pediatric patients.

Because the increasing number of subgroups interferes with the statistical power of clinical trials, multinational centralized research projects must be established. Hope exists for new alternative treatment options, such as targeted therapy or even individualized tumor treatment. Radiation therapy techniques have also significantly improved, and adverse effects are expected to be further decreased. Some tumor subgroups will no longer require irradiation. Certain subgroups of medulloblastomas and ependymomas can currently be successfully treated without radiation therapy [111]. The need for tumor tissue biopsy will probably be replaced by refined MRI techniques, liquid biopsies, or NGS-based diagnostics in the future.

Currently, however, (pediatric) neurosurgeons must ensure maximally safe tumor resection and sufficient tumor tissue acquisition for adequate molecular-histological classification and further research on targeted therapies.

## Figures and Tables

**Table 1:** Tumor subgroups (extract and simplification) according to the 5th WHO classification with genes/molecular profiles [4,7]. By permission of Oxford University Press.

Tumor Type	Subtype	Histopathological Classification	Molecular Information, Key Diagnostic Genes, Molecules Pathways	CNS WHO Grade 1–4
**Gliomas**				
Pediatric-type diffuse low grade gliomas	Diffuse astrocytoma, MYB- or MYBL1-altered	Diffuse glioma with low proliferation	MYB, MYBL1	1–2
	Angiocentric glioma	astrocytic or oligodendroglial morphology	MYB	
	Polymorphous low-grade neuroepithelial tumor of the young		BRAF, FGFR family	
	Diffuse low grade glioma, MAPK pathway altered		FGFR1, BRAF	
Pediatric-type diffuse high grade gliomas	Diffuse midline glioma, H3 K27-altered	glial morphology	H3 K27, TP53, ACVR1, PDGFRA, EGFR, EZHIP	3–4
	Diffuse hemispheric glioma, H3 G34-mutant		H3 G34, TP53, ATRX	
	Diffuse pediatric-type high-grade glioma, H3-wildtype and IDH-wildtype		IDH-wildtype, H3-wildtype, PDGFRA, MYCN, EGFR, (methylome)	
	Infant-type hemispheric glioma		NTRK family, ALK, ROS, MET	
Circumscribed astrocytic gliomas	Pilocytic astrocytoma	astrocytoma	KIAA 1549-BRAF, BRAF, NF1	1–3
	High grade astrocytoma with piloid features		BRAF, NF1, ATRX, CDKN2A/B (methylome)	
	Pleomorphic xanthoastrocytoma		BRAF, CDKN2A/B	
	Subependymal giant cell astrocytoma		TSC1, TSC2	
	Chordoid glioma		PRKCA	
	Astroblastoma, MN1-altered		MN1	
**Ependymomas**				
Supratentorial ependymoma	Supratentorial ependymoma, ZFTA fusion-positive	ependymoma	ZFTA, RELA, YAP1, MAML2	2–3
	Supratentorial ependymoma, YAP1 fusion-positive			
Posterior fossa ependymoma	Posterior fossa ependymoma, group PFA		H3, K27me3, EZHIP (methylome)	
	Posterior fossa ependymoma, group PFB			
Spinal ependymoma	Spinal ependymoma, MYCN-amplified		NF2, MYCN	2–3
Myxopapillary ependymoma	Myxopapillary ependymoma			2
Subependymoma	Subependymoma	subependymoma		1
**Embryonal brain tumors**				
Medulloblastoma	Medulloblastoma, molecularly defined	medulloblastoma		4
	Medulloblastoma, wingless (WNT)-activated		CTNNB1, APC	
	Medulloblastoma, sonic hedghog (SHH)-activated and TP53-wildtyp		TP53, PTCH1, SUFU, SMO, MYCN, GLI2	
	Medulloblastoma, SHH-activated and TP53-mutant			
	Medulloblastoma, non-WNT/non-SHH: group 3 and group 4		MYC, MYCN, PRDM6, KDM6A	
Other CNS embryonal tumors	Atypical teratoid/rhabdoid tumor (ATRT)	embryonal morphology	SMARCB1, SMARCA4	4
	Cribriform neuroepithelial tumor			
	Embryonal tumor with multilayered rosettes (ETMR)		C19MC, DICER1	4
	CNS neuroblastoma, FOXR2-activated		FOXR2	
	CNS tumor with BCOR internal tandem duplication		BCOR	
	CNS embryonal tumor			

CNS: central nervous system, SHH: Sonic Hedgehog, WHO: World Health Organization, WNT: Wingless.

**Table 2:** Tumor subgroups according to the 5th WHO classification and recommended treatment (no claim to completeness, data for rare tumor entities only base on case series/reports) [2,4,7,49]. By permission of Oxford University Press.

Tumor Type	Subtype	Surgery/Watch and Wait	Radiotherapy	Chemotherapy	Others (See also Section 4)
**Gliomas**					
Pediatric-type diffuse low grade gliomas (see also Section 5.3)	Diffuse astrocytoma, MYB- or MYBL1-altered [50]	+ GTR / watch and wait	+ when not resectable	+ when not resectable and to delay radiotherapy	(+) BRAF, MEK 1/2 inhibitors and others
	Angiocentric glioma				
	Polymorphous low-grade neuroepithelial tumor of the young				
	Diffuse low grade glioma, MAPK pathway altered				
Pediatric-type diffuse high grade gliomas (see also Section 5.3)	Diffuse midline glioma, H3 K27-altered [51]	+ Biopsy if possible and in ethically approved clinical study [52]	+ Radiotherapy	+ (e.g., Temolozomide with CCNU) (+) trials-based chemotherapy (when possible to improve resectability) [52]	(+) BRAF, MEK, MAPK inhibitors, mTOR Inhibitors, histone deactylase inhibitors (for K27M mutations), and others
	Diffuse hemispheric glioma, H3 G34-mutant [53]	+ GTR, STR, Biopsy [53,54]	+ Radiotherapy	(+) chemotherapy [53,54] role unclear	
	Diffuse pediatric-type high-grade glioma, H3-wildtype and IDH-wildtype				
	Infant-type hemispheric glioma [54]				(+) TRK Inhibitor [54]
Circumscribed astrocytic gliomas (see also Section 5.3)	Pilocytic astrocytoma	+ GTR	− only when not resectable and older than 3-5 years of age		
	High grade astrocytoma with piloid features [55]	+ GTR/STR/Biopsy [55]	(+)	+ Chemotherapy (e.g., temozolomid) [55]	
	Pleomorphic xanthoastrocytoma [56]	+ GTR	(+) unclear only when not resectable and older than 3-5 years of age	(+) unclear, might have a benefit when not resectable [56]	(+) unclear BRAF, MEK inhibitors [56]
	Subependymal giant cell astrocytoma [57,58]	(+) GTR more and more replaced by mTOR [57,58]	-	-	+ mTOR inhibitor (for reducing tumor growth) [57,58]
**Ependymomas** (see also Section 5.4)					
Supratentorial ependymoma	Supratentorial ependymoma, ZFTA fusion-positive Supratentorial ependymoma, YAP1 fusion-positive	+ GTR + second look/repeated surgery when residual [26]	+ Local radiotherapy (when older than 12-18 months of age) [26] + Craniospinal irradiation in case of CSF or spinal dissemination boost [26]	debatable, option when younger than 12-18 months of age [26]	
Posterior fossa ependymoma	Posterior fossa ependymoma, group PFA Posterior fossa ependymoma, group PFB				PFB or YAP fusion possibly without radiotherapy
Spinal ependymoma	Spinal ependymoma, MYCN-amplified	+ GTR [26]	+ Only in incomplete resection or WHO 3 [26]	-	
Myxopapillary ependymoma	Myxopapillary ependymoma	+ GTR [26]	evaluation in incomplete resection [26]		
**Embryonal brain tumors**					
Medulloblastoma (see also Section 5.2)	Medulloblastoma wingless (WNT)-activated	+ GTR	+ Craniospinal + boost to the tumor bed	+ multiagent chemotherapy, Depending on subclassification (+/− intrathecal methotrexate, reduced therapy may not be required in WNT tumors)	(+) different targets: SHH: e.g., SMO-inhibitors
	Medulloblastoma sonic hedghog (SHH)-activated and TP53-wildtyp				
	Medulloblastoma SHH-activated and TP53-mutant				
	Medulloblastoma non-WNT/non-SHH: group 3 and group 4				
Other CNS embryonal tumors	Atypical teratoid/rhabdoid tumor (ATRT) [59]	+ GTR	+ Radiotherapy [59]	(+) unclear: multiagent and high dose chemotherapy [59]	(+) AURK, CDK4/6 and other Inhibitors [59]
	Embryonal tumor with multilayered rosettes (ETMR) [60]	+ GTR [60]	+ focal or craniospinal Radiotherapy [60]	(+) chemotherapy [60]	

Surgery: + indicated/standard treatment, (+) additional/no clear survival benefit, (−) not recommended.CNS: central nervous system, GTR: gross total resection if feasible, STR: subtotal resection, SHH: Sonic Hedgehog, WHO: World Health Organization, WNT: Wingless.

## Data Availability

Not applicable.
